# Targeting of tumor endothelial cells combining 2 Gy/day of X-ray with Everolimus is the effective modality for overcoming clinically relevant radioresistant tumors

**DOI:** 10.1002/cam4.185

**Published:** 2014-01-27

**Authors:** Yoshikazu Kuwahara, Miyuki Mori, Shuji Kitahara, Motoi Fukumoto, Taichi Ezaki, Shiro Mori, Seishi Echigo, Yasuhito Ohkubo, Manabu Fukumoto

**Affiliations:** 1 Department of Pathology, Institute of Development, Aging and Cancer, Tohoku UniversitySendai, Miyagi, 980-8575, Japan; 2 Department of Dentistry and Oral surgery, National Hospital Tokyo Medical CenterTokyo, 152-8902, Japan; 3 Department of Anatomy and Developmental Biology, Tokyo Women's Medical UniversityTokyo, 162-8666, Japan; 4 Department of Oral and Maxillofacial Surgery, Tohoku University HospitalSendai, Miyagi, 980-8575, Japan; 5 Department of Oral Medicine and Surgery, Graduate School of Dentistry, Tohoku UniversitySendai, Miyagi, 980-8575, Japan; 6 Department of Radiopharmacy, Tohoku Pharmaceutical UniversitySendai, Miyagi, 981-8558, Japan

**Keywords:** Clinically relevant radioresistant, Everolimus, fractionated radiation, thrombus, tumor endothelial cells

## Abstract

Radiotherapy is widely used to treat cancer because it has the advantage of physically and functionally conserving the affected organ. To improve radiotherapy and investigate the molecular mechanisms of cellular radioresistance, we established a clinically relevant radioresistant (CRR) cell line, SAS-R, from SAS cells. SAS-R cells continue to proliferate when exposed to fractionated radiation (FR) of 2 Gy/day for more than 30 days in vitro. A xenograft tumor model of SAS-R was also resistant to 2 Gy/day of X-rays for 30 days. The density of blood vessels in SAS-R tumors was higher than in SAS tumors. Everolimus, a mammalian target of rapamycin (mTOR) inhibitor, sensitized microvascular endothelial cells to radiation, but failed to radiosensitize SAS and SAS-R cells in vitro. Everolimus with FR markedly reduced SAS and SAS-R tumor volumes. Additionally, the apoptosis of endothelial cells (ECs) increased in SAS-R tumor tissues when both Everolimus and radiation were administered. Both CD34-positive and tomato lectin-positive blood vessel densities in SAS-R tumor tissues decreased remarkably after the Everolimus and radiation treatment. Everolimus-induced apoptosis of vascular ECs in response to radiation was also followed by thrombus formation that leads to tumor necrosis. We conclude that FR combined with Everolimus may be an effective modality to overcome radioresistant tumors via targeting tumor ECs.

## Introduction

Radiotherapy (RT) is one of the major therapeutic modalities for cancer treatment, especially for early-stage cancers because of its excellent tumor control, preservation of normal tissues, and fewer systemic influences 1. The general protocol for RT consists of daily exposure to fractionated radiation (FR) of 2-Gy X-rays for 5–7 weeks. The underlying principle behind fractionated RT is that normal tissue cells repair damaged DNA more efficiently than cancer cells because the normal cells proliferate slower. Tumors receive a large total dose from multiple FR and can sometimes recur with radioresistance, eventually leading to failure of RT 2. Several mechanisms have been implicated in acquiring radioresistance, including the selection of intrinsic radioresistant cells in a heterogeneous tumor population and the induction of radioresistance mutations 3,4. However, the precise mechanisms behind acquired radioresistance remain unclear. Elucidating the molecular mechanisms for acquiring radioresistance is essential for the development of more effective RT with FR.

To improve the efficacy of RT for tumors, a combination treatment of radiation and an anticancer drug, that is, chemoradiotherapy, is widely used. DNA damage can result directly from radiation or indirectly from reactive oxygen species 5. These effects are not limited to only tumor cells but also microvascular endothelial cells (ECs) in the tumor stroma 6. Therefore, radiosensitivity of solid tumors is determined not only by intrinsic tumor cell factors but also by the microvascular network that provides oxygen to the tumor.

The development of the tumor microvascular network by angiogenic processes is necessary for tumor growth and metastasis. Tumor cells produce growth factors that stimulate the proliferation and migration of ECs, which forms new blood vessels within the tumor 7. The irregular architecture and high permeability of tumor microvessels cause blood flow heterogeneity, resulting in high interstitial fluid pressure and hypoxic tumor areas. These hypoxic tumor areas are resistant to RT 8. Consequently, antiangiogenic factors such as angiostatin, vascular endothelial growth factor (VEGF), VEGF receptor inhibitors, and epidermal growth factor receptor (EGFR) inhibitors have been used in combination with RT. These modalities show at least an additive effect for tumor growth control 9–11. However, it is controversial whether a combination of radiation with antiangiogenic therapy improves tumor growth control or not.

Radioresistance has been associated with the activation of distinct intracellular signaling pathways in tumor cells in response to radiation 12. In particular, radiation induces tumor cell proliferation in a dose-dependent manner between 0.5 and 2 Gy by activating the phosphoinositide 3-kinase (PI3K)/Akt pathway, most likely by stimulating EGFRs on tumor cells 13,14. Rapamycin is a macrolide originally found as an antifungal agent and is now recognized as having anticancer and immunosuppressive properties 15. The mammalian target of rapamycin (mTOR) is a downstream effector of the PI3K/Akt pathway. mTOR controls translation of specific mRNA transcripts that encode for cell cycle progression and cell proliferation proteins 16. Therefore, mTOR has been becoming an important target of a new line of anticancer drugs. CCI-779, a rapamycin derivative, exhibits significant anticancer activity in various tumor cell lines 17. Everolimus, a derivative of rapamycin formulated for daily oral administration, affects cancer cells in numerous ways such as decreasing cell proliferation and increasing apoptosis and autophagic cell death 18. Recently, Everolimus has also been reported to have antiangiogenic properties 19. Therefore, Everolimus can be expected to act as a radiosensitizer in human solid tumors.

We have previously established a clinically relevant radioresistant (CRR) cell line, SAS-R, derived from SAS cells 20. SAS-R cells continue to proliferate with daily X-ray exposures of 2 Gy for more than 30 days in vitro. Xenografted SAS-R tumors were resistant to X-rays when exposed to 2 Gy/day of FR for 30 days, while SAS tumors were not. In this study, we established a CRR tumor model that is a unique and powerful tool for understanding and conquering radioresistant tumors. Our in vitro study indicated that hyperinduction of autophagy radiosensitizes CRR cells 21. Therefore, we hypothesized that enhancing autophagy in CRR cells by Everolimus would also effectively control radioresistant tumors. In this study, we used this CRR tumor model to determine whether the combination treatment of radiation and Everolimus could effectively overcome the CRR phenotype and to analyze the molecular mechanisms underlining tumor radioresistance in vivo.

## Materials and Methods

### Cell culture

The human oral squamous cell carcinoma cell line (SAS) and human cervical cancer cell line (HeLa) were obtained from the Cell Resource Center for Biomedical Research, Institute of Development, Aging, and Cancer (IDAC). CRR SAS-R and HeLa-R cell lines were established from SAS and HeLa cells by gradually exposing cells to increasing doses of X-rays, as previously described 20. To maintain the CRR phenotype, SAS-R and HeLa-R cells were exposed to 2 Gy of X-rays every 24 h, and the cumulated dose was more than 1,000 Gy. Cells were maintained in Roswell Park Memorial Institute (RPMI) 1640 medium (Nacalai Tesque Inc., Kyoto, Japan) supplemented with 5% fetal bovine serum (Gibco Invitrogen Corp., Carlsbad, CA) in a humidified atmosphere at 37°C with 5% CO_2_. Human dermal microvascular endothelial cells (HMVECs) were purchased from Kurabo Industries, Ltd. (Osaka, Japan). HMVECs were cultured according to the manufacturer's protocol. X-ray irradiation was performed at the dose of 1 Gy/min in a 150-KVp X-ray generator (MBR-1520R; Hitachi, Tokyo, Japan) with a total filtration of 0.5 mm aluminum and 0.1 mm copper.

### Cell proliferation assay

The effect of Everolimus on cell proliferation was analyzed using Cell Count Reagent SF (Nacalai Tesqe Inc., Kyoto, Japan), according to the manufacturer's protocol. Briefly, 5 × 10^3^ cells/well in 100 *μ*L of medium was cultured in 96-well microtiter plates (BD-Falcon Biosciences, Lexington, TN). After 24 h, Everolimus was added to cell culture, and plates were incubated for another 48 h. Subsequently, 10 *μ*L of Cell Count Reagent were added to each well. After incubating for 2 h, cell proliferation was assessed by measuring absorbance at 450 nm using a MULTISKAN JX (Thermo Fisher Scientific K.K., Yokohama, Japan). Everolimus (RAD001) and its vehicle were kindly provided by Novartis Pharma AG for Biochemical Research (Basel, Switzerland).

### Modified high-density survival (MHDS) assay

The MHDS assay was performed as previously described 20. Briefly, cells were treated with 3 nmol/L Everolimus for 3 h and seeded in 25 cm^2^ flasks at a density of 5×10^5^ cells/flask. After 48 h, the cells were exposed to various doses of X-rays and incubated for 72 h. Next, 1/10 of the cells in each flask were seeded into a new 25 cm^2^ flask, and the total number of cells in each flask was counted using the trypan blue dye exclusion test after another 72 h incubation.

### Western blotting

Activating mTOR enhances protein translation by phosphorylating eukaryotic initiation factor 4E-binding protein 1 (4E-BP1) and the 70-kDa S6 kinase 1 (S6K1) 22. Therefore, S6 phosphorylation (p-S6) can be used as a biomarker for mTOR activation 23. The day before the experiments, 5 × 10^5^ cells were seeded into a 25 cm^2^ flask (BD-Falcon Biosciences). For Everolimus treatment in vitro, cells were incubated in medium containing 3 nmol/L Everolimus for 1 or 3 h.

To prepare whole cell lysate, cells were washed twice with ice-cold phosphate-buffered saline (PBS) and then resuspended in a lysis buffer containing 25 mmol/L sodium phosphate (pH 7.4), 500 mmol/L NaCl, 1 mmol/L ethylenediaminetetraacetic acid (EDTA, pH 8.0), 0.5% Triton-X 100, 0.1% glycerol, 5 mmol/L MgCl_2_, 1 mmol/L dithiothreitol (DTT), 1 mmol/L phenylmethylsulfonyl fluoride (PMSF), and protease inhibitor cocktail (Nacalai Tesqe Inc.). Cells were centrifuged for 20 min at 4°C, and supernatants were stored at −80°C until use. The whole cell lysate (20 *μ*g) was electrophoresed in sodium dodecyl sulfate (SDS) polyacrylamide gel and electroblotted onto a polyvinylidene fluoride membrane. After blocking with 5% skim milk overnight at 4°C, membranes were incubated with primary antibodies at 4°C overnight and then with horseradish peroxidase-conjugated secondary antibodies (Nichirei Biosciences Inc., Tokyo, Japan). Anti-mTOR, anti-phospho-mTOR, anticleaved caspase-3, and anti-LC3 antibody were purchased from Cell Signaling Technology Japan, K.K. (Tokyo, Japan) and anti-p62/SQSTM1 antibody from MBL Co., Ltd. (Nagaya, Japan). Bands were visualized using Chemi-Lumi One L western blotting substrate (Nacalai Tesqe Inc.).

### Enzyme-linked immunosorbent assay (ELISA)

The levels of VEGF-A secreted by cells was estimated by the VEGF ELISA assay system (GE Healthcare UK Ltd., Buckinghamshire, UK), according to the manufacturer's protocol. Briefly, 5 × 10^5^ cells were seeded in a 60 mm^2^ dish, and culture medium that was conditioned for 24 h was used for the ELISA assay.

### The effect of FR with Everolimus on xenograft tumors

Male BALB/c nude mice that were 4 weeks old were used for all experiments. All experimental protocols were reviewed by the Committee on the Ethics of Animal Experiments, Tohoku University and were carried out in accordance with the University's Guidelines for Animal Experiments. Exponentially growing cells (1 × 10^7^ cells in 250 *μ*L saline) were subcutaneously injected into the backs of nude mice. When tumor volumes reached about 150 mm^3^, experiments were started (day 0). Two days before starting FR (2 Gy/day), Everolimus (5 mg/kg) was administered orally daily with a vehicle by Novartis. Only vehicle was given to the controls. Mice were given FR every 24 h for 30 days. Except for the tumor regions, the bodies of the mice were protected from radiation by a lead shield. Tumor volumes were estimated using a caliper and calculated according to the following formula: tumor volume = 0.5 × length × width × height 24.

### Histological analysis and immunohistochemistry of SAS and SAS-R tumors

Mice with tumors were perfused with 4% paraformaldehyde (PFA) via the left ventricle of the heart 24 h after the last treatment. The excised tumor tissues were postfixed in 10% neutral-buffered formalin overnight and 4-*μ*m thick paraffin-embedded sections were prepared. The sections were stained with hematoxylin and eosin. Pycnotic cells were scored as apoptotic cells, and the total number of cells and apoptotic cells per field (×200) was scored. At least 200 cells per field were counted in tumors from three different mice.

Tissue sections were heated in antigen retrieval solution (pH 9.0; Nacalai Tesqe Inc.) by microwave for 10 min and cooled to room temperature. After washing with PBS, the slides were treated with 3% H_2_O_2_-methanol for 10 min at room temperature to quench endogenous peroxidase activity. Tissue sections were incubated with 5% skim milk for 60 min at room temperature in a humidity chamber to reduce nonspecific staining. The sections were incubated overnight at 4°C with primary antibodies to detect either CD34 (Abcam Inc., Cambridge, MA), p-S6 (Cell Signaling Technology Japan, K.K.), p62 (Cell Signaling Technology Japan, K.K.), single-stranded DNA (Dako Japan, Inc., Tokyo, Japan), or VEGF-A (Santa Cruz Biotechnology Inc., Santa Cruz, CA). After several washes with PBS, the specimens were incubated for 60 min at room temperature with the corresponding secondary antibodies (Nichirei Biosciences Inc.). Signals were visualized by incubating with 3,3′-diaminobenzidine followed by counterstaining with hematoxylin. Sections were photographed with a BZ-Analyzer (Keyence, Osaka, Japan).

### Analysis of microvessel density (MVD)

To label blood vessels, 1.25 × 10^6^ SAS or SAS-R tumor cells were transplanted into the dorsal right flank of BALB/c nude mice. When tumor diameters reached 2 mm, treatments were started. Four days after initiating the treatments (day 1), we used intravascular perfusion with fluorescent tomato lectin to label all blood vessels that had a patent blood supply. Briefly, anesthetized mice were injected intravenously with 100 *μ*L fluorescein isothiocyanate (FITC)-conjugated tomato lectin (lycopersicon esculentum lectin; 1 mg/mL; Vector Laboratories, Burlingame, CA). After 10 min, mice were perfused. The tumors were then excised and postfixed in 10% neutral-buffered formalin overnight, and 10-*μ*m thick cryosections were prepared. After air-drying, the cryosections were rehydrated in PBS and then incubated with 1% Block Ace blocking solution (Dainippon Seiyaku, Tokyo, Japan) to reduce nonspecific background staining. Double immunostaining was performed overnight at 4°C in humidified chambers using a primary antibodies against the mouse endothelial cell marker CD31 (BD Pharmingen, San Diego, CA) and type IV collagen (Cosmo Bio Co. Ltd., Tokyo, Japan). After several washes with PBS, the specimens were incubated with a Cy3-conjugated secondary antibody (Jackson ImmunoResearch, West Grove, PA) for 45 min at room temperature. Specimens were mounted with Vectashield (Vector Laboratories Inc.) and analyzed using a confocal laser scanning microscope (Leica, Wetzlar, Germany).

### Transmission electron microscopy (TEM)

Mice were perfused with 4% PFA in PBS. Their tumor tissues were removed and cut into small blocks (approximately 1 mm^3^ in size) and immersed in 2% glutaraldehyde in 0.1 mol/L PBS for at least 2 h. The blocks were treated with 1% OsO_4_, dehydrated with a graded series of ethanol and propylene oxide, and embedded in epoxy resin. Ultrathin sections (70 nm thick) were cut, stained with lead citrate, and examined using a H-7000 electron microscope (Hitachi).

### Statistical analysis

The data were analyzed by Student's *t*-test. Results are expressed as mean ± standard deviation (SD) of three independent experiments.

## Results

### Establishing the in vivo CRR tumor model

**Figure 1:**
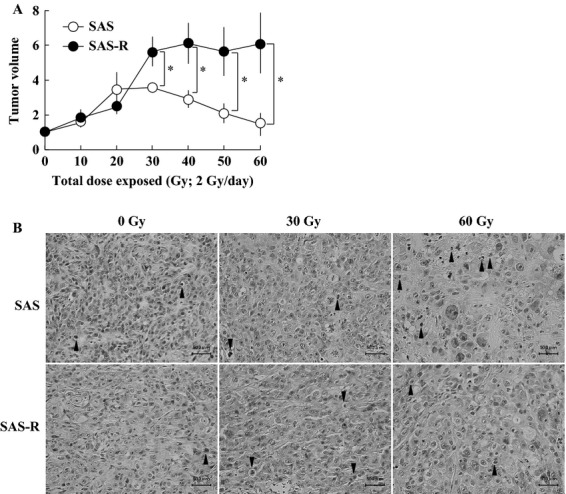
(A) Establishment of a clinically relevant radioresistant tumor model. Exponentially growing SAS and SAS-R cells were subcutaneously injected into the dorsal flank of nude mice. When tumor volumes reached about 150 mm^3^ (day 0), mice were exposed to FR with 2 Gy/day of X-rays for 30 days. Mean ± SD of three independent mice are shown. *P < 0.01. (B) Histological analysis of SAS and SAS-R tumors exposed to 2 Gy/day of fractionated X-rays. Connective tissues were more abundant in SAS-R tumors than in SAS tumors. Arrow heads, pyknotic cells. FR, fractionated radiation.

Although SAS-R cells retained the CRR phenotype in vitro 20, it was unknown whether SAS-R cells maintained this CRR phenotype in vivo. After treatment began, SAS tumors continued to grow until 10 days after starting FR (total dose 20 Gy) (Fig. 1A). From the 20th day of FR, tumor volume gradually decreased. When the total dose reached 60 Gy (30 days), the SAS tumor volume returned to that at the beginning of this experiment. In contrast, the volume of SAS-R tumors continued to increase when exposed to 2 Gy/day of FR. On day 30 (60 Gy), the SAS-R tumor volume had increased sixfold.

Histological analysis of SAS and SAS-R tumors revealed that connective tissues were more abundant in SAS-R tumors than in SAS tumors. The frequency of pyknotic cells was not significantly different between SAS (3.4 ± 0.8%) and SAS-R tumors (3.0 ± 1.5%) without FR (Fig. 1B). The frequency of pyknotic cells and the amount of connective tissues tended to increase after 15 days of FR in SAS tumors, but not in SAS-R tumors. After 30 days of FR (60 Gy), the frequency of pyknotic cells was significantly higher in SAS tumors (14.1 ± 0.8%) compared to SAS-R tumors (7.7 ± 2.5%) (*P* < 0.01).

We performed Ki-67 staining to examine the tumor cells that were proliferatively active when exposed to FR from X-rays. Without irradiation, the frequency of Ki-67-positive cells was 84.1 ± 4.6% and 68.5 ± 2.4% in SAS and SAS-R tumors, respectively. After 15 days of FR (30 Gy), the frequency of Ki-67-positive cells was 47.3 ± 13.8% and 65.9 ± 14.8% in SAS and SAS-R tumors, respectively. After 30 days of FR (60 Gy), the frequency of Ki-67-positive cells significantly decreased to 43.9 ± 9.6% in SAS tumors (*P *< 0.05), but not in SAS-R tumors (67.8 ± 7.4%).

### MVD of SAS and SAS-R tumors

**Figure 2:**
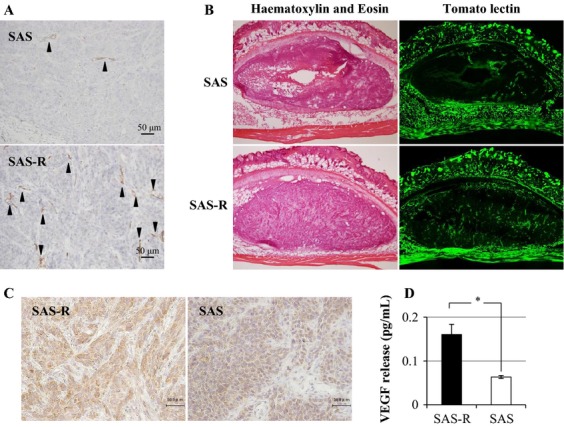
(A) Immunohistochemical analysis of CD34-positive blood vessels (arrow heads) in xenografted tumors without any treatments. CD34 immunostaining confirmed that MVD in SAS-R tumors was higher than in SAS tumors. (B) Blood vessel density determined by tomato lectin labeling in SAS and SAS-R tumors. Tomato lectin labeling of the luminal endothelial cell surface showed that MVD with blood flow was higher in SAS-R tumors compared to in SAS tumors. (C) Immunohistochemical staining of VEGF-A. SAS-R tumor cells had stronger immunostaining for VEGF-A than SAS tumor cells. (D) VEGF-A production from SAS and SAS-R cells in vitro determined by ELISA. Twofold secretion of VEGF-A from SAS-R cells compared to SAS was detected. *P < 0.01. MVD, microvessel density; VEGF, vascular endothelial growth factor.

Connective tissues were more abundant in SAS-R tumors than in SAS tumors (Fig. 1B). This observation prompted us to examine MVD in SAS and SAS-R tumors. CD34 immunostaining confirmed that MVD in SAS-R tumors was higher than in SAS tumors (Fig. 2A). Tomato lectin labeling of the luminal EC surface showed that MVD with blood flow was higher in SAS-R tumors compared to in SAS tumors (Fig. 2B). This suggested that angiogenesis was activated in SAS-R tumors. MVD was also higher in HeLa-R tumors than in HeLa tumors (data not shown).

We further studied why MVD was higher in CRR tumors than in parental tumors. SAS-R tumor cells had stronger immunostaining for VEGF-A than SAS tumor cells (Fig. 2C). This observation was confirmed by ELISA of in vitro cultures (Fig. 2D). Subsequently, we analyzed the effect of the antiangiogenesis drug Everolimus on the radiosensitivity of CRR tumors.

### Everolimus inhibited mTOR phosphorylation in vitro and in vivo

**Figure 3:**
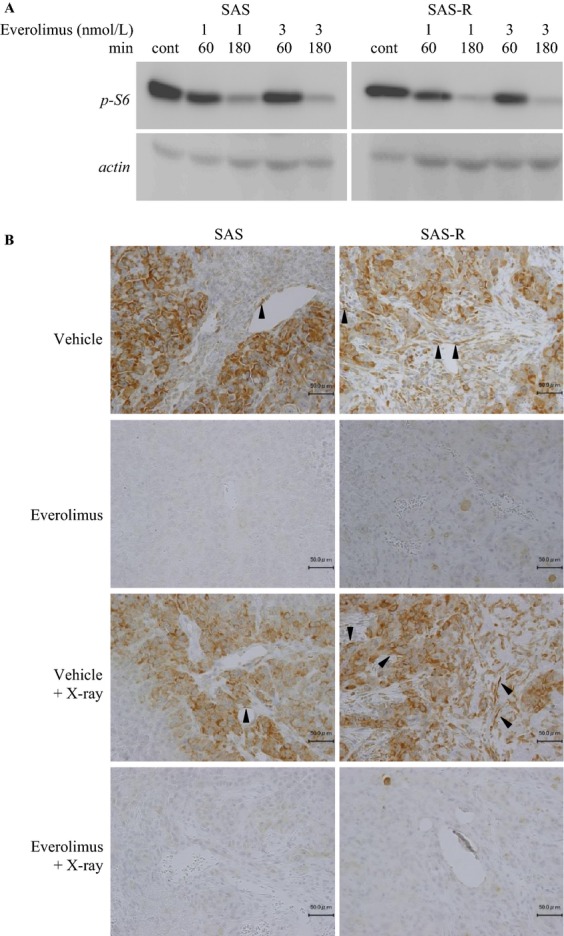
(A) Western bloting of p-S6 with or without Everolimus. Everolimus inhibited p-S6, a downstream target of mTOR, in SAS and SAS-R cells. Cells were treated with 1 and 3 nmol/L Everolimus, and cell lysates were collected at 0, 60, and 180 min. (B) Immunohistochemical analysis of p-S6. Three days after each treatment, p-S6 expression was analyzed. p-S6 was detected in endothelial (arrowheads) and tumor cells in SAS and SAS-R tumors. Everolimus completely suppressed p-S6 expression, even after radiation exposure, in endothelial and tumor cells of SAS and SAS-R. mTOR, mammalian target of rapamycin.

SAS and SAS-R cells were similarly positive for p-S6, indicating that the mTOR pathway was activated in both SAS and SAS-R cells in vitro (Fig. 3A). Everolimus completely inhibited the amount of p-S6 in SAS and SAS-R cells in vitro.

Without Everolimus, SAS and SAS-R tumor cells were strongly positive for p-S6 (Fig. 3B). However, p-S6-positive ECs were more abundant in SAS-R tumors than in SAS tumors. Everolimus suppressed p-S6 expression in ECs. Three days after starting FR, the amount of p-S6 positive ECs increased in SAS-R, but not SAS tumors. This increase was also completely suppressed by Everolimus.

### Everolimus with FR inhibited tumor growth of SAS-R

**Figure 4:**
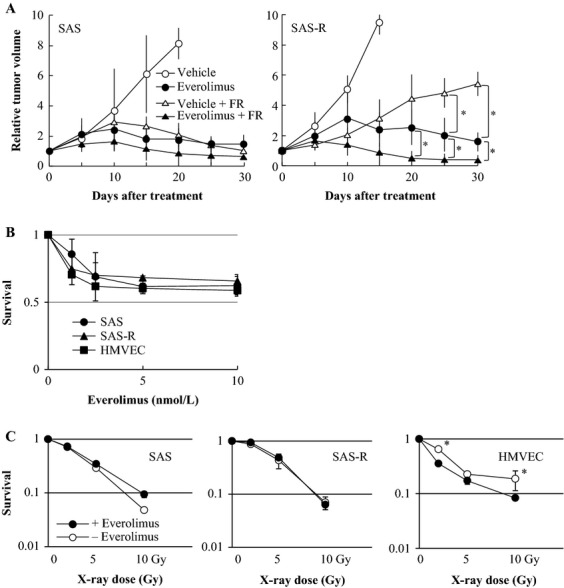
(A) Fractionated radiation (2 Gy/day) with Everolimus suppressed tumor growth in SAS and SAS-R tumors. Everolimus with 2 Gy/day of X-rays effectively reduced tumor volumes of SAS and SAS-R tumors. Mean ± SD of three independent mice. (B) Everolimus sensitivity of SAS and SAS-R cells and HMVECs determined by a cell proliferation assay. Everolimus sensitivity was similar among all cell lines examined. Mean ± SD in triplicate. (C) Radiation sensitivity of SAS and SAS-R cells and HMVECs with or without Everolimus. Everolimus significantly radiosensitized HMVECs, but not SAS or SAS-R cells, even at exposure to 2 Gy of X-rays. Mean ± SD in triplicate *P < 0.05. FR, fractionated radiation; HMVEC, Human dermal microvascular endothelial cells.

Until 10 days after each treatment, the tumor volume of SAS increased; thereafter, it gradually decreased in all the treatment groups with FR, FR with Everolimus, and Everolimus alone during the experimental period. The volume of SAS tumors with vehicle continuously increased (Fig. 4A). On the other hand, until 10 days after Everolimus treatment the tumor volume of SAS-R increased followed by gradual decrease, but the tumor volume remained larger than that at the beginning. SAS-R tumors grew by 5 days of the treatment with Everolimus and FR. Thereafter, the tumor volume shrunk. On day 30, the volume of SAS-R tumors treated with FR and Everolimus was smaller than the initial volume (day 0). The most effective treatment for the control of SAS-R tumors was the combination of FR with Everolimus.

### Everolimus-targeted ECs

We performed cell survival assay in vitro to clarify which is the main radiosensitizing target of Everolimus, tumor cells, or ECs. The survival rate was not significantly different among SAS cells, SAS-R cells, and HMVECs after treatment with Everolimus (Fig. 4B). Everolimus significantly radiosensitized HMVECs, but not SAS and SAS-R cells, even at X-ray exposures of just 2 Gy in vitro (Fig. 4C). This suggested that Everolimus targeted the ECs of blood vessels in tumors rather than tumor cells.

**Figure 5:**
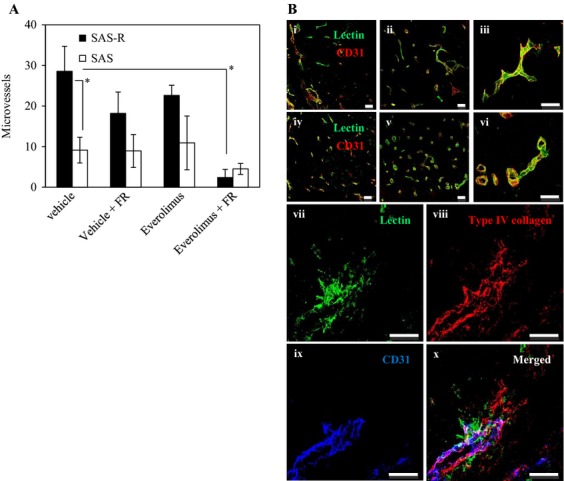
(A) CD34-positive microvessels 3 days after the treatment of fractionated radiation (FR) and Everolimus. Everolimus significantly decreased microvessel density in SAS-R tumors when combined with FR. Mean ± SD of three independent mice. (B) Everolimus-induced morphological changes of microvessels. (i–vi) Tomato lectin labeling of blood-circulating vessels followed by CD31 immunostaining. After 3 days of treatment, the discrepancy between tomato lectin and CD31 staining decreased in SAS-R tumors treated with Everolimus. In SAS-R tumors without Everolimus, tumor vessels acquired wide lumens. In contrast, lumen diameter did not decrease in SAS tumors. (i) SAS tumor without Everolimus. (ii) SAS tumor with Everolimus. (iii) SAS tumor with Everolimus. (iv) SAS-R tumor without Everolimus. (v) SAS-R tumor with Everolimus. (vi) SAS-R tumor with Everolimus. (vii–x) Representative vasculature ruptures in SAS-R tumors after FR and Everolimus treatment. (vii) A collapsed vessel with leaking tomato lectin satining. (viii) Type IV collagen staining showing the rupture of the basal membrane of vessels. (ix) CD31 immunohistochemistry. (v) vii–ix merged. Scale bur: 50 *μ*m. FR, fractionated radiation.

We further investigated MVD by counting CD34-positive blood vessels with or without radiation or Everolimus (Fig. 5A). Before treatment, MVD was significantly higher in SAS-R tumors than in SAS tumors (*P *< 0.01). Everolimus slightly, but not significantly, decreased MVD in SAS-R tumors. FR did not influence MVD in both SAS and SAS-R tumors. However, combining radiation and Everolimus significantly decreased MVD in SAS-R tumors when compared with radiation or Everolimus alone. Radiation with Everolimus also slightly decreased MVD in SAS tumors when compared with other treatment groups; however, this was not statistically significant.

### Everolimus-induced morphological changes in microvessels

The distribution of both tomato lectin and CD31 was identical, indicating that the tumor blood vessels detected on a microscope were functionally active (Fig. 5B). Everolimus did not change the morphology of blood vessels in SAS tumors (Fig. 5B i–iii). However, in SAS-R tumors Everolimus treatment changed the lumen of tumor vessels from slit-like to round tubular (Fig. 5B iv–vi). Many disrupted vessels, accompanied by tomato lectin leakage and erythrocyte extravasation, were observed in SAS-R tumors after treatment with FR and Everolimus (Fig. 5B vii–x).

### Formation of thrombi in tumors treated with Everolimus

**Figure 6:**
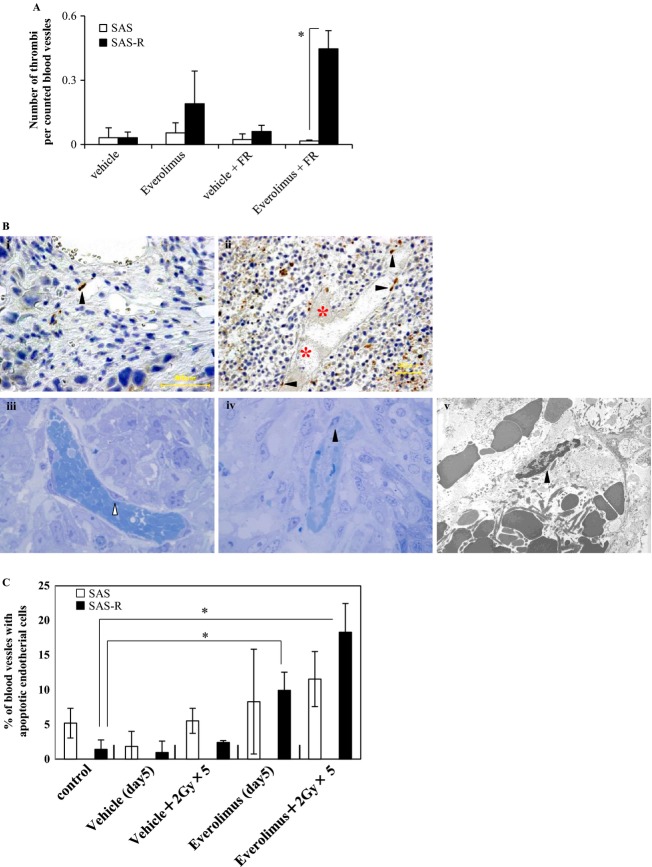
(A) The number of the thrombus per counted blood vessels. The highest density was observed in SAS-R tumors treated with fractionated radiation (FR) and Everolimus. Mean ± SD in triplicate. *P < 0.01. (B-i and ii) Immunohistochemical detection of single-strand DNA-positive cells. Black arrow heads, single-strand DNA-positive endothelial cells. red asterisk, thrombosis. (B*-*i) SAS tumor 24 h after exposure to 5 days of FR (2 Gy/day) with Everolimus. (B*-*ii) SAS-R tumor 24 h after exposure to 5 days of FR (2 Gy/day) with Everolimus. Thrombosis was frequently found. (B*-*iii) Most endothelial cells of blood vessels surrounding the surviving tumor area had no nucleus with condensed chromatin in SAS-R tumors 3 days after starting Everolimus and FR treatment. White arrow head, endothelial cell. (B*-*iv, v) Some apoptotic tumor cells (e.g., with condensed chromatin) near the central necrosis area were found by vessels with apoptotic endothelial cells containing old thrombosis. Black arrow heads, apoptotic endothelial cells. (iii, iv) Toluidine blue staining. ×1250 magnification. (v) ×4000 magnification. (C) Frequencies of blood vessels with apoptotic endothelial cells. Even Everolimus alone significantly induced endothelial cell apoptosis in SAS-R tumors, however, these endothelial cell apoptosis were remarkably reinforced by Everolimus combining with FR. In contrast to SAS-R tumor tissues, significant induction of apoptotic endothelial cells in SAS tumors was not observed by Everolimus compared with control, even after combining with FR. *P < 0.01. FR, fractionated radiation.

We examined thrombus density (the number of thrombi per counted blood vessels). Thrombus density was higher in the Everolimus group than in controls (Fig. 6A). The highest thrombus density occurred in SAS-R tumors treated with FR and Everolimus.

Subsequently, we examined the death of ECs in SAS and SAS-R tumors by the detection of single-strand DNA. Prominent EC death was not detected in vessels surrounding the surviving tumor area in SAS tumors 24 h after exposure to 5 days of FR with Everolimus (Fig. 6B i). In contrast, many blood vessels with EC death and thrombosis were found in SAS-R tumors 24 h after exposure to 5 days of FR with Everolimus (Fig. 6B ii). We further studied the mode of EC death induced by Everolimus with FR in SAS-R tumors on an electron microscope. ECs in vessels surrounding the surviving tumor area had no nuclei with condensed chromatin 3 days after starting Everolimus and FR treatment (Fig. 6B iii). In contrast, near the central necrosis area, ECs with condensed chromatin were found with old thrombosis (Fig. 6B iv–v), suggesting that EC death induced by Everolimus with FR was apoptosis.

Even Everolimus alone induced significant EC apoptosis in SAS-R tumors compared with the control group; however, apoptosis was remarkably reinforced by the combination with FR (Fig. 6C). In contrast to SAS-R tumors, significant induction of apoptosis in SAS tumors was not detected by Everolimus even with FR compared with control.

## Discussion

In this study, we established a CRR tumor model that can function as a unique and powerful tool for understanding radioresistant tumors. Our previous in vitro study indicated that induction of autophagy radiosensitizes CRR cells 21. Therefore, we hypothesized that enhancing autophagy would also effectively control radioresistant tumors in vivo. As expected, autophagic cell death was not remarkable in SAS-R tumors after FR. Therefore, we intended to induce autophagic cell death in SAS-R tumors using Everolimus to overcome radioresistance 25. Everolimus is a derivative of rapamycin, a specific mTORC1 and angiogenesis inhibitor and an autophagy inducer. Although the size of SAS-R tumors began to decrease dramatically within 10 days from starting Everolimus and FR combination treatment, the induction of autophagic cell death was not apparently observed within this period (data not shown). This suggests that the effect of Everolimus on reducing tumor volume is not primarily due to the induction of autophagic tumor cell death. Cell survival assay in this study suggested that the primary target of Everolimus in tumor tissues is vascular ECs rather than tumor cells. Immunohistological analysis of p-S6 revealed that radiation exposure activated the mTOR pathway of ECs in SAS-R tumors compared to SAS tumors. These results prompted us to examine the effect of Everolimus on vascular ECs.

Inhibiting mTOR results in an accumulation of G1-phase cells and apoptosis 26. In this study, Everolimus did not affect proliferation or radiosensitivity of both SAS and SAS-R cells in vitro. However, Everolimus reduced tumor volumes for both SAS and SAS-R tumors and radiosensitized SAS-R tumors. Everolimus sensitized HMVECs in vitro and also induced apoptosis in vascular ECs of SAS-R. Therefore, we thought that decreased volume of Everolimus-treated tumors was a consequence of impaired blood supply by thrombosis formation due to the induction of apoptosis in ECs. SAS-R cells deceased faster than SAS cells under serum depletion or 2-deoxy-d-glucose treatment with FR in vitro (data not shown). This indicated that SAS-R cells require more nutrients and/or oxygen than SAS cells. Therefore, the effect of Everolimus is more prominent in SAS-R tumors than in SAS tumors. Studies of the relationship between cellular metabolic activity and radiosensitivity are now underway in our laboratory.

Inhibiting the PI3K/Akt/mTOR pathway enhances radiation-induced destruction of tumor blood vessels 27. mTOR is a downstream target of Akt, and we hypothesized that mTOR signaling was activated in ECs of SAS-R tumors. Interestingly, radiation exposure increased the number of p-S6 positive ECs, especially in SAS-R tumors, and p-S6 positivity was completely suppressed by Everolimus. Therefore, the increased radiosensitivity of ECs in SAS-R tumors via Everolimus was efficient for overcoming radioresistance.

Rapamycin has a strong antiangiogenic effect by interfering with VEGF-mediated pathways in ECs 28. Interestingly, the effect of Everolimus on tumor growth control results from promoting thrombosis in SAS-R tumors rather than inhibiting VEGF expression in tumor cells. A previous study indicated that rapamycin enhances the delay in tumor growths when used with radiation in mouse xenograft models 29. This delay in tumor growth results from the effects of rapamycin on thrombosis 30. In this study, mTOR signaling was activated in both tumor and vascular ECs of SAS-R and SAS tumors. Everolimus radiosensitized HMVECs but not SAS or SAS-R tumor cells in vitro. This suggests that mTOR is essential for endothelium survival in SAS-R tumors. Therefore, we propose that mTOR inhibitors sensitize vascular ECs to radiation.

Tumor-associated fibroblasts (TAFs) play an important role in many aspects of tumor physiology. As part of the tumor microenvironment, TAFs transmit different molecular signals to cancer cells and other cell types present in the tumor stroma. This dynamic signaling has been shown to contribute to tumor initiation, progression, and metastasis 31. In SAS-R tumors, type IV collagen was more abundant than in SAS tumors (data not shown). This suggests that the characteristics of TAF in SAS-R tumors may differ from those in SAS tumors. This may contribute to the radioresistance of SAS-R tumors. The MVD of SAS-R tumors was also higher than that of SAS tumors in this study. Matsuda et al. demonstrated that tumor ECs had a stronger response to VEGF and higher VEGF receptor-1 and -2 expression than normal ECs 32. We think that the increased VEGF released from SAS-R cells affected the MVD differences between SAS-R and SAS tumors.

Although Everolimus changed the shape of blood vessels from slit-like to round and tubular in SAS-R tumors, this change was not observed in SAS tumors. This suggests that ECs in SAS-R tumors may have different sensitivity to chemotherapy and RT compared to SAS tumors. Lane et al. reported that Everolimus inhibits the proliferation of VEGF-stimulated human ECs and impairs VEGF release from tumor cells 24. This suggests that the reduction in tumor volume in SAS-R tumors after Everolimus and radiation treatment is attributed to the targeting of tumor vessels rather than tumor cells. Combining X-ray FR (2 Gy) and Everolimus (10 ng/mL) significantly suppressed cell proliferation in L3.6pl pancreatic cancer cells and CT-26 colon cancer cells in vitro 18. However, in this study, Everolimus alone failed to radiosensitize SAS-R cells in vitro. In contrast, Everolimus radiosensitized HMVECs. Guba et al. have demonstrated that the potent antiangiogenic activity of rapamycin can be attributed to two factors: reduced VEGF production by tumor cells and the inhibition of VEGF-induced proliferation of endotherial cells. Blood vessel function can also be impaired by rapamycin via stimulating thrombosis within tumor microvasculature 30. Tomato lectin staining indicated significant vascularity reductions and vasculature disruptions in SAS-R tumors. The vascular density of SAS-R tumors treated with Everolimus and radiation reduced more than that treated with Everolimus or radiation alone. We think that the reduction in SAS-R tumor volume induced by combination of Everolimus and radiation is attributed to enhanced apoptosis of ECs. We conclude that radiation combined with Everolimus may be effective to overcome tumor radioresistance via targeting vascular ECs in radioresistant tumors.
